# Genetic variation in nuclear and mitochondrial markers supports a large sex difference in lifetime reproductive skew in a lekking species

**DOI:** 10.1002/ece3.1188

**Published:** 2014-09-03

**Authors:** Yvonne I Verkuil, Cedric Juillet, David B Lank, Fredrik Widemo, Theunis Piersma

**Affiliations:** 1 Animal Ecology Group, Centre for Ecological and Evolutionary Studies, University of GroningenPO Box 11103, Groningen, 9700 CC, the Netherlands; 2 Department of Natural History, Royal Ontario Museum100 Queen’s Park Crescent, Toronto, M5S 2C6, Ontario, Canada; 3 Department of Biological Sciences, Simon Fraser UniversityBurnaby, V5A 1S6, British Columbia, Canada; 4 Department of Fish, Wildlife and Environmental Studies, Swedish University of Agricultural SciencesUmeå, SE- 901 83, Sweden; 5 Department of Marine Ecology, NIOZ Royal Netherlands Institute for Sea ResearchPO Box 59, Den Burg, Texel, 1790 AB, the Netherlands

**Keywords:** Effective population size, *Philomachus pugnax*, ruff, sexual selection, shorebirds

## Abstract

Sex differences in skews of vertebrate lifetime reproductive success are difficult to measure directly. Evolutionary histories of differential skew should be detectable in the genome. For example, male-biased skew should reduce variation in the biparentally inherited genome relative to the maternally inherited genome. We tested this approach in lek-breeding ruff (Class Aves, *Philomachus pugnax*) by comparing genetic variation of nuclear microsatellites (*θ*_*n*_; biparental) versus mitochondrial D-loop sequences (*θ*_*m*_; maternal), and conversion to comparable nuclear (*N*_*e*_) and female (*N*_*ef*_) effective population size using published ranges of mutation rates for each marker (*μ*). We provide a Bayesian method to calculate *N*_*e*_ (*θ*_*n*_ = 4*N*_*e*_*μ*_*n*_) and *N*_*ef*_ (*θ*_*m*_ = 2*N*_*ef*_*μ*_*m*_) using 95% credible intervals (CI) of *θ*_*n*_ and *θ*_*m*_ as informative priors, and accounting for uncertainty in *μ*. In 96 male ruffs from one population, *N*_*e*_ was 97% (79–100%) lower than expected under random mating in an ideal population, where *N*_*e*_:*N*_*ef*_ = 2. This substantially lower autosomal variation represents the first genomic support of strong male reproductive skew in a lekking species.

## Introduction

In a lek system, a small number of males obtain most of the copulations, and therefore the distribution of mating success is strongly positively skewed with a mode near zero (Höglund and Alatalo 1995). An issue of interest in studies of lek mating systems is the extent to which seasonal or apparent skews in mating success ultimately produce a comparable skew in lifetime reproductive success (LRS) (Mackenzie et al. 1995; Kokko et al. 1998, 1999). Variance in LRS may be smaller than expected because mating skews at leks are not necessarily mirrored by a skew in paternity because of off-lek copulations. This is the case in the lekking Houbara bustard (*Chlamydotis undulata undulate*) (Lesobre et al. 2010). In addition, mating skews can be highly age dependent (e.g., Gibson and Guinness 1980; McDonald 1989), multiple paternity of offspring can reduce the mating variance in polygynous species (Webster et al. 1995), and trade-offs against survivorship may occur.

Directly measuring the skew in LRS requires comprehensive paternity analyses for at least one generation, which is difficult or impossible in many study systems (but see e.g., DuVal 2012). However, an evolutionary history of sex differences in skew in LRS should be visible in the genome. Strong sexual selection should result in a strongly reduced effective population size for the selected sex; for male-lekking species, reduced neutral genetic diversity should occur in paternally only inherited genes (Chesser and Baker 1996). In a recent paper, approximately a 10% reduction in neutral genetic variation on the Z chromosome was found in two polygynous shorebird species (Corl and Ellegren 2012). Because lekking is generally associated with extreme polygyny, an even stronger reduction in variation is expected in lekking species. In the lekking Gunnison sage-grouse (*Centrocercus minimus*), the simulated reduction in effective population size due to male mating skew was at least 23%, while accounting for the variance in seasonal reproductive success in females, which was almost as high as in males due to a high rate of nest failure (Stiver et al. 2008). In the lek-breeding European treefrog (*Hyla arborea*), no sex differences in effective population size were found; in this species, a negative effect of the mating system was obscured by the much stronger effect of delayed maturity in both sexes (Broquet et al. 2009).

Here, we revisit the empirical support for the predicted reduction in paternally transmitted genetic variation in a lekking species (for predictions see: Chesser and Baker 1996; Charlesworth 2009; Corl and Ellegren 2012). The test case is the ruff (*Philomachus pugnax*), which has a well-described lek mating system. The species has (1) highly ornamented males that are much larger than the nonornamented females, (2) three genetically based alternative male morphs, and (3) strong annual reproductive skews in males (Hogan-Warburg 1966; van Rhijn 1991; Lank et al. 1995, 2013; Widemo 1998; Jukema and Piersma 2006), all of which points toward strong sexual selection on males (Shuster and Wade 2003). However, in studies of direct paternity of ruffs, many offspring could not be assigned to fathers (Lank et al. 2002; Thuman and Griffith 2005), indicating that copulations took place with nonsampled males at other leks or off-lek (Lank and Smith 1987; van Rhijn 1991), potentially reducing the extent of variance in mating success relative to that assumed from counting copulations at leks (see Widemo and Owens 1995). Potential negative trade-offs between mating success and longevity are undocumented. Therefore, the magnitude of skew in the lifetime reproductive success of males remains unknown.

We developed a method that can be generally applied to detect sex differences in skews in lifetime reproductive success in birds, when, or assuming that ecological knowledge is available to cautiously explore other deviations from equilibrium conditions, such as sex differences in dispersal, recruitment, survival, and selection. Our Bayesian approach compares genetic variation of biparentally inherited autosomal genes with genetic variation of a maternally only inherited mitochondrial gene and estimates the likelihood to reject the random mating hypothesis under which autosomal variation is larger than mitochondrial variation. The Bayesian framework accounts for variation and uncertainty in mutation rates using a range of published mutation rates for each of the genetic markers.

## Materials and Methods

### Sampling

Ruffs have slight population structure across a breeding range that extends from Western Europe to eastern Siberia (Verkuil et al. 2012). To avoid potential effects of population substructure, we confined the present analysis to samples collected at one breeding site, in Sweden. Blood samples were taken from the brachial vein of 96 territorial male ruffs captured with cannon nets between 1990 and 2001 on Gotland, Sweden (57°10′N, 18°20′E) (Widemo 1997; Thuman 2003). Blood samples were stored in 95% ethanol, and DNA was isolated by standard phenol–chloroform or Chelex extractions and stored at −20°C.

### Choice of genetic markers

For this study, highly variable markers were used that are generally applied in population genetic studies: microsatellites or simple sequence repeats (SSRs), and sequences of the mitochondrial D-loop or control region. These mutate fast and are therefore adequate archives of recent genetic evolution (Avise 2004). Importantly, data on genetic variation collected with these markers are available for many study organisms. The disadvantage is that marker-specific mutation rates (*μ*) have to be used to convert genetic variation to comparable units, and estimates of *μ* often have large uncertainties. Our method explores the use of these markers while accounting for the uncertainty in *μ*.

### Biparental loci

The biparentally transmitted genes used to genotype the 96 males were seven polymorphic autosomal microsatellites loci (nDNA): ruff1, ruff6, ruff10, ruff12, and ruff50 are published loci cloned from ruffs (Thuman et al. 2002), and SNIPE B2 (Sæther et al. 2007) and M2 (L. Wennerberg, pers. comm.). PCR profiles and details on fragment analysis, and the assignment of alleles can be found in Thuman et al. (2002) and Sæther et al. (2007). Standard diversity indices were obtained with the program Migrate (Beerli 2006).

### Maternal locus

The maternally inherited locus used in this study is the control region (CR) of the mitochondrial genome (mtDNA). A 512 nt segment of domain I and II of the CR was amplified and sequenced with forward primer L141 (5′-TCCATTAATCTACAACCGGGCT) and reverse primer PropR (5′-AATACCAGCTTTGGGAGTTGG). Ruffs have a duplicated CR with high sequence similarity between copies (Verkuil et al. 2010). By anchoring the primer PropR in the tRNA^Pro^, only CR1 was targeted. The amplification profile used was 2 min denaturation at 95°C, followed by 36 cycles of 94°C for 45 s, 53–57°C for 45 s, 72°C for 1.30 min, followed by a final 7 min elongation at 72°C. PCR products were gel-purified and prepared for sequencing using BigDye Terminal Cycle Sequencing reagents according to the manufacturer’s instructions (Applied Biosystems, Foster City, CA, USA) and sequenced on an ABI 3100 automated sequencer. Sequences were edited and aligned in MEGA 3.1 (Kumar et al. 2004), and standard diversity indices were obtained with ARLEQUIN, version 3.11 (Excoffier and Schneider 2005).

### Estimation of Θ with coalescent analysis

To obtain the effective population size from the genetic data, the following equations need to be applied,

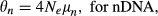
(1)

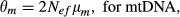
(2)where *θ*_*n*_ and *θ*_*m*_ are the respective nuclear and mitochondrial genetic variance parameters (i.e., sequence diversity that evolved under a species-specific effective population size and mutation rate), *N*_*e*_ and *N*_*ef*_ are biparental and female-transmitted only effective populations sizes, and *μ*_*n*_ and *μ*_*m*_ the marker-scaled mutation rates (Hartl and Clark 1997: Pp. 121–127). The factor four in equation 1 accounts for the ploidy difference with mtDNA and the estimation over two branches. The factor two in equation 2 accounts for the estimation of *θ* over two branches of the ancestral tree.

Estimates of the variance parameters *θ*_*n*_ and *θ*_*m*_ were obtained by coalescent analysis (Beerli and Felsenstein 1999), using the program Migrate-n version 3.5.1 (Beerli 2006). The genotypes and haplotypes were considered to come from a genetically uniform population, because we used males from a single breeding population. For microsatellites, the Bayesian Brownian motion model was used, which is a continuous modification of the discrete stepwise mutation model (Kuhner 2006). After initial runs (with various random seed numbers) to determine likely priors for *θ*_*n*_ leading to unimodal posterior distributions, one final long run was executed with a uniform prior distribution for *θ* of [0;100]. After a burn-in of 20,000 steps, the parameter space was sampled by one adaptively heated chain, sampling 200,000 trees, with an increment of 100; a total of 300 replicates were run, meaning that in total 6 billion parameter values were visited. Settings were adjusted to allow substitution rates to vary among loci (P. Beerli, pers. comm.).

For mtDNA sequences, Bayesian interference was also used. The substitution rate was assumed to be constant between sites and the transition/transversion bias was set to *R* = 33 (R was 33 in a larger sample of all breeding birds, see Verkuil et al. (2012)). After initial runs to test for convergence under different priors, a uniform prior distribution of [0.0;0.1] was set for theta. One final run was performed with a burn-in of 10,000 and a static heating scheme of four temperatures, sampling 10 million trees with an increment of 100; consequently, 1 billion parameter values were visited.

### Modeling *N*_*e*_:*N*_*ef*_ under uncertainty in both mutation rates and genetic variance parameters

The quantity *N*_*e*_:*N*_*ef*_ is provided by equations 1 and 2,

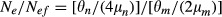
(3)

However, the uncertainty in the parameters *θ* and *μ* can substantially affect the estimation of *N*_*e*_ and *N*_*ef*_ from genetic data. To estimate *N*_*e*_:*N*_*ef*_, we used OpenBUGS, a Bayesian analyses dedicated software that makes the MCMC method easily accessible (Spiegelhalter et al. 2010). For calculation of *N*_*e*_, a generation time of 3 years was assumed (Colwell 2010). In the computation of *N*_*e*_:*N*_*ef*_, we incorporated the uncertainty of published mutation rates (*μ*_*n*_ and *μ*_*m*_) and the genetic variance parameters (*θ*_*n*_ and *θ*_*m*_), estimated from the data. Instead of using single fixed values, we defined two uniform prior distributions, one for *θ*_*n*_ and one for *θ*_*m*_. The uniform distributions were defined over a range of values that included 95% of their respective posterior distribution obtained by coalescent analysis of the genetic data (*U*[10.0;16.5] and *U*[0.01;0.05], respectively, see Results).

For the mutation rates *μ*_*n*_ and *μ*_*m*_, we defined, a priori, the range of possible mutation rates according to the literature ([1 × 10^−5^;1 × 10^−3^] and [1 × 10^−8^;1 × 10^−7^], respectively). This means that the high variability of mutation rates, as reported in the literature, is the starting point of the Bayesian analysis. To determine the ranges of mutation rates, we used the following information. In vertebrates, the typical mutation rate for microsatellites is 1 × 10^−4^ substitutions/locus/generation (Ellegren 2000; Whittaker et al. 2003). Birds have fewer microsatellites than mammals, but characteristics such as length, allele dispersion, and range of allele sizes do not vary between birds and mammals, indicating that mutation rates might be comparable (Neff and Gross 2001). Recently, fast microsatellite mutation rates have been reported in one Falconiformes and three Passeriformes, ranging between 3.0 × 10^−3^ and 1.3 × 10^−2^ (Brohede et al. 2002; Beck et al. 2003; Ortego et al. 2008; Masters et al. 2011); the variation in mutation rates between these four studies strongly correlated with median allele spans ([24–140] bp). Our microsatellites had a median allele span of 36 bp (Verkuil et al. 2012). We therefore assumed an upper boundary for *μ*_*n*_ of 1 × 10^−3^. To stay conservative, a lower boundary of 1 × 10^−5^ was assumed.

For mtDNA, a *μ*_*m*_ of 2–15% per Ma was assumed, which translates into [2 × 10^−8^; 1.5 × 10^−7^] substitutions/locus/year (using control region sequence of 512 nt). This range includes observed values for shorebirds for domain I of the control region (Wenink and Baker 1996) and also the lower mutation rate in domain II (Buehler and Baker 2005). In the results, the term “CI” corresponds to the credible interval of estimates.

## Results

### Genotypic data

Ninety-six individuals were successfully genotyped at seven microsatellite loci. None of the loci had null-alleles, significant linkage disequilibrium, or significant deviations from Hardy–Weinberg equilibrium (see Thuman et al. 2002). The number of alleles per locus was 9, 14, 10, 5, 12, 20, and 15, respectively. The expected heterozygosity (*H*_*exp*_) was 0.695 (0.419–0.845).

Eighteen individuals were successfully sequenced for the 512-bp segment of the mitochondrial control region. The segment had sixteen polymorphic sites (15 transitions and 1 transversion), and across the samples 14 different haplotypes were detected (GenBank under Accession Nos HQ171099-HQ171161). The gene diversity (*H*_*s*_) was 0.954 ± 0.039, and the average nucleotide diversity was 0.007 ± 0.004.

### Estimates of theta

The coalescent run to estimate theta from microsatellites had an effective MCMC sample size of 609 million and returned an unimodal posterior distribution with a global estimate of *θ*_*n*_ of 13.23 (95% CI 10.2–16.6). For mtDNA, the effective MCMC sample size was 5.5 million, with resulted in a unimodal posterior distribution, and a most probable estimate of *θ*_*m*_ of 0.0238 (95% CI 0.012–0.048).

### Ratio of biparental to maternal effective population size

Including the 95% CIs of the estimates of *θ*_*n*_ and *θ*_*m*_, the ratio *N*_*e*_:*N*_*ef*_ was 0.068 (SD = 0.168, 95% CI 0.004–0.425), assuming prior uniform distributions for *μ*_*m*_ and *μ*_*n*_ as given in the Methods. This means that in ruffs, nuclear genetic variation is 97% (range: 79–100%) lower than expected under random mating in an ideal population, where *N*_*e*_:*N*_*ef*_ = 2.

## Discussion

The 97% reduction in nuclear genetic variation is empirical support for the assumed stronger interindividual variance in lifetime reproductive success (LRS) of male ruffs relative to females. This result confirms the intuition of previous field researchers who observed the large investments by some males in dominating the lek (Hogan-Warburg 1966; van Rhijn 1983, 1991). Males may display so intensively that they refrain from eating and drinking, and lose mass throughout the breeding season (Widemo 1998; Bachman and Widemo 1999). Our study suggests that for the winners, male competition indeed does pay off in a high LRS. Annual and lifetime reproductive skew probably exists within all three male morphs (independents, satellites and faeders, see Jukema and Piersma 2006; Lank et al. 2013), but the skew among independents will drive the reduction of genetic variation, because they comprise ca. 80–85% of the population (Lank et al. 1995; Widemo 1998). Strong skews are hypothesized to facilitate invasion of alternative reproductive strategies (Shuster and Wade 2003), as clearly has occurred in this case. The results show that in ruffs sex-specific fitness variation (sexual selection) has been strong and stable long enough for the nuclear genetic variation to be much lower than the mitochondrial genetic variation inherited through the female line.

Our approach to evaluate the degree of difference in genetic variation between the nuclear and mitochondrial genome through estimating the effective population size (*N*_*e*_) can be applied more generally. The degree of difference in *N*_*e*_ between the two genomes will summarize longer-term mating skews between the sexes in any species. However, knowledge of the ecology of the species is a prerequisite, because sex differences in dispersal, recruitment, survival, and selection may obscure the effect of the mating system (see Chesser and Baker 1996; Barton 2000; Waples 2010). We will use ruffs to illustrate the possible effects of sex differences in ecology on the reduction in *N*_*e*_.

Stronger male than female dispersal will reduce *N*_*e*_. For example in an extreme case, 90% of the dispersal could take place through males, which would reduce *N*_*e*_ by as much as 65% (expected *N*_*e*_*:N*_*ef*_ changes from 2 to 0.7, see the mammal model of Chesser and Baker 1996). Theoretically, lekking bird species are assumed to have female-biased dispersal (Greenwood and Harvey 1982; Chesser and Baker 1996), but empirical data are inconclusive on this prediction (see Lebigre et al. 2008; Martín et al. 2008). In ruffs, female natal philopatry is extremely low (Andersen 1948, 1951); adults of both sexes show large geographic flexibility in migration routes (Rakhimberdiev et al. 2011). At the same time, adult territorial males in our study population show substantial lek site fidelity between years (Widemo 1997) and were never observed to shift to another breeding area once established (Lif 2002; F. Widemo, unpublished data). Therefore, it is unlikely that ruffs have strongly male-biased dispersal.

Adult sex ratio can also influence *N*_*e*_. Ruffs have an assumed skewed adult sex ratio of ca. 40% males to 60% females (OAG Münster 1996; Zwarts et al. 2009), which may result from a sex bias in recruitment (Jaatinen et al. 2010), as found in other polygynous birds (Donald 2007; see Kokko and Jennions 2008). This sex ratio bias would lead to a reduction in *N*_*e*_ of 35%, even under random mating (expected *N*_*e*_:*N*_*ef*_ changes from 2 to 1.3).

In principle, *N*_*e*_:*N*_*ef*_ can also be influenced by differential selection regimes in the two genomes and/or the Hill-Robertson interference; mtDNA does generally not recombine and is therefore susceptible to genetic sweeps reducing variation (Marais 2007). This would lead to an underestimate of *N*_*ef*_. Also, if genetic diversity was not at mutation/drift equilibrium (e.g., the species is recovering from a severe population bottleneck), microsatellites would have higher diversity than mitochondrial genome because mutation rate rather than *N*_*e*_ would control the speed of recovery. As we found relatively high variation in mtDNA, in ruffs neither process can be likely responsible for the observed low *N*_*e*_:*N*_*ef*_ ratio.

We conclude that in ruffs, the observed reduction in *N*_*e*_ of 97% (range: 79–100%) is stronger than can be accounted for by ecological differences between males and females, and primarily results from the larger variance in LRS in males than in females, which is congruent with the predicted *N*_*e*_*:N*_*ef*_ under the lek model of Chesser and Baker (1996). This is not the first study assessing the reduction of genetic variation in a lekking species (e.g., Stiver et al. 2008; Broquet et al. 2009; Corl and Ellegren 2012), but it is the first to detect a reduction. Ruffs were included in a comparison of genetic diversity at autosomal and Z-linked introns of monogamous and polygynous shorebird species (Corl and Ellegren 2012), but had few variable sites compared with the other species, and the authors of this earlier study considered the results inconclusive for ruff. We showed that a long-term mating skew can be genetically assessed in ruffs using microsatellites and the mitochondrial control region, which have a higher mutation rates than the nuclear introns used in the earlier study. The uncertainty in mutation rate of these markers should be accounted for with methodology such as we presented. Using our approach, existing and future genotyping datasets from other species may be used to characterize their mating systems. The method will be most useful if information on other potential biasing ecological factors is available, but mating patterns themselves cannot be directly assessed.

## Data Accessibility

DNA sequences: GenBank Accession Nos: HQ171099-HQ171161.
